# Bifurcation and hybrid control of a discrete eco-epidemiological model with Holling type-III

**DOI:** 10.1371/journal.pone.0304171

**Published:** 2024-07-18

**Authors:** Lizhi Fei, Hengmin Lv, Heping Wang

**Affiliations:** 1 College of Science, Nanchang Institute of Technology, Nanchang, Jiangxi, P. R. China; 2 Key Laboratory of Engineering Mathematics and Advanced Computing of Nanchang Institute of Technology, Nanchang, Jiangxi, P. R. China; 3 College of Horticulture, Nanjing Agricultural University, Nanjing, Jiangsu, P. R. China; University of Milano–Bicocca: Universita degli Studi di Milano-Bicocca, ITALY

## Abstract

In this paper, a three dimensional discrete eco-epidemiological model with Holling type-III functional response is proposed. Boundedness of the solutions of the system is analyzed. Existence condition and stability of all fixed points are discussed for the proposed model. Furthermore, we obtained the transcritical bifurcation surfaces of the system by bifurcation theory. Based on the explicit criteria for the Neimark Sacker bifurcation and flip bifurcation, we obtained that the system undergoes these two types of bifurcations at the positive fixed point. Then we apply a hybrid control strategy that based on both parameter perturbation and a state feedback strategy to control the Neimark-Sacker bifurcation. Finally, some numerical simulations are carried out to support the analytical results.

## Introduction

Eco-epidemiology is considered to be a relatively new branch of mathematical biology that studies both ecological and epidemiological issues simultaneously. In 1986, Anderson and May [1] firstly considered disease factor in the predator-prey system. They coupled the epidemiological model developed by Kermack and Mckendrick [2] to a Lotka-Volterra predator-prey model [3, 4]. Chattopadhyay and Arino [5] coined the term ‘eco-epidemiology’ [6, 7], and proposed a predator-prey epidemiological model with disease spreading in prey, and one of its simplified models takes the form as follows

$$\begin{array}{c} \left\{ \begin{array}{c} \frac{dS}{t}=rS(1-\frac{S+I}{K})-\beta SI,\\ \frac{dI}{t}=-cI+\beta SI-pIY,\\ \frac{dY}{t}=-dY+qpIY, \end{array}\right. \end{array}$$ (1)

where *S*, *I*, *Y* represent the populations of susceptible prey species, infected prey species and predator species, respectively. In 2001, Chattopadhyay et al [8] studied the practical problem of Pelicans at risk in the Salton Sea based on this model.

The Salton Sea, the largest inland water body in California, USA, is a eutrophic salty lake. In the summer, the weather reaches 128 degrees Fahrenheit and the water evaporates very quickly [9]. This process increases the salinity of the Salton Sea and reduces oxygen levels, as saltwater is more difficult to combine with oxygen than freshwater. Four types of fishes are very common in Salton Sea, namely Tilapia, Corvina, Croaker, and Sargo. Among them, Tilapia is the most abundant because of its amazing reproductive rate [10]. The Salton Sea is the main habitat for many migratory birds such as pelicans, but thousands of water birds (most of which are pelicans) and fish have died. The real cause of this is not yet clear, but there is growing evidence pointing to toxic algal blooms. Algal blooms grow and die very quickly, and in doing so, it draws oxygen from seawater. A lack of oxygen in the tissues of infected fish can lead to outbreaks of botulism, and the later stages of the disease cause the infected fish to rise closer to the surface in search of oxygen. As reported by US Geological Survey National Wildlife Health Center, many white pelicans died from botulism type C between 1978 and 2003 [11]. In 1996, over 8500 white Pelicans died due to infection with type C botulism in the Salton Sea. Millions of dead or sick fish can transmit botulism to Pelicans and other migratory birds that feed on fish. As pelicans prey on vulnerable fish, the ingestion of botulinum has lead to the development of avian botulism [12]. In the past, many research has been done considering various aspects of interaction and interrelation of Tilapia fish, botulism type C, and Pelican birds. System (1) and its extensions have been investigated under different conditions and functional response. Such as the eco-epidemiological predator-prey model with diseases in the prey [13–17], with a disease in the predator [18–21], with disease in both populations [22–24], with delay [25, 26] and with Holling type functional response [27–30].

However, these studies mostly focus on continuous time system, and rarely involve discrete time system. It is also important to consider discrete-time models. Firstly, discretization of continuous time model is the basis of obtaining numerical approximate solution [31]. Secondly, due to the fact that statistical data of epidemics are collected in discrete time, it is more convenient and accurate to describe epidemics with a discrete-time model [32, 33]. Thirdly, many species, such as monocarpic plants, and semelparous animals have independent and non-overlapping generations, and their births occur during the regular breeding season. Their interactions are described using difference equations or discrete-time models [34]. Moreover, even a single-species discrete-time model can exhibit bifurcation, chaos, and more complex dynamic behaviors. Recently, discrete-time models have received more and more attention, see [35–40] and the references cited therein. Whereas, these studies mainly focus on two-dimensional discrete-time systems, with relatively few studies on the dynamics of three-dimensional discrete-time systems, and even fewer studies on the dynamics of three-dimensional discrete-time eco-epidemiological systems.

**Fig 1:**
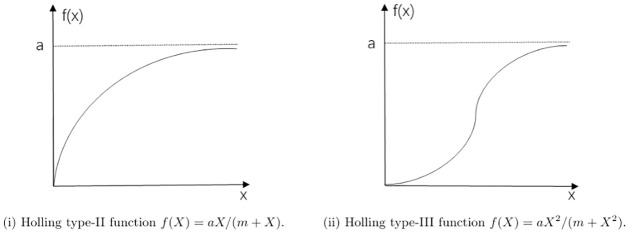
Holling type functional responses function.

Lately, several works related to discrete-time eco-epidemiological models have appeared in the literature. In [41], the authors considered the discretization system of system (1) with ratio-dependent Michaelis-Menten functional response. Then, in [42], the authors considered a discrete system with saturated incidence rate based on paper [41], and obtained the stability, bifurcation and chaos of the system. In [43], the authors considered the discretization system of system (1) with Holling type-II functional response. But there has been limited study on three-dimensional discrete-time systems with Holling type-III functional response, due to its highly non-linear nature. This motivated us to consider a discrete-time eco-epidemiological model with Holling type-III functional response incorporating disease in prey to study the interaction between Tilapia and the Pelican. In this article, the existence and stability of fixed points, bifurcation analysis and a hybrid control strategy are discussed. Through this article, we make the following assumptions.

(A1) The disease is spread among the prey population Tilapia only and the disease is not genetically inherited. The infected population does not recover or become immune. The prey is divided into two classes, susceptible *x* and infective *y*. The disease incidence rate adopts bilinear incidence rate *βxy*, and *β* is the transmission coefficient.
(A2) In the absence of disease, the prey population Tilapia grows according to the logistic law with intrinsic growth rate *r* and carrying capacity *K*. Only susceptible prey is capable of reproducing, the infective preys cannot produce offsprings due to the disease or by predation before having the possibility of reproducing. However, as infected prey still consumes resources, it still contributes to carrying capacity. Thus, the growth rate for the prey is *rx*(1 − (*x* + *y*)/*K*).
(A3) The predator population Pelican consumes only the infected Tilapia. This is conform to the fact that the infected individuals are less active and can be more easily captured [8].
(A4) The predator consumes prey following Holling type-III functional response. In fact, if the predator actively seeks out large concentration of prey the Holling type-III function *f*(*X*) = *aX*^2^/(*m* + *X*^2^) is more appropriate, where *a* is the maximum predation rate and *m* is half saturation constant. Since the slope of this function goes to zero for small values of *X*(f’(0)=0), it may be suspected that the food chain will be destabilized if prey concentration becomes too small [44]. However, after a certain value of *X*, the predators increase their feeding rates until some saturation level is reached (*f*(*X*) → *a* when *X* → ∞). Holling type III functional response is mostly used when the number of predator encounters the prey population with a very lower amount due to unavailability of prey but when the prey becomes available then the response behaves like the Holling II type functional response (*f*(*X*) = *aX*/(*m* + *X*)) [45]. Although both Holling II and III functional responses are approaching an asymptote, the former is decelerating and the latter is sigmoid [46]. Holling [46] suggested that the type II responses are characteristic of predators, which have no learning ability or when give only one type of prey for which to search, whereas type III responses are characteristic of vertebrate predators where switching and learning are more common. We show the Holling type-II and III function in Fig 1.

Under the above assumptions, we formulate the following discrete-time eco-epidemiological model

$$\left\{ \begin{array}{c} x_{n+1}=x_{n}+rx_{n}(1-\frac{x_{n}+y_{n}}{K})-\beta x_{n}y_{n},\\ y_{n+1}=y_{n}+\beta x_{n}y_{n}-\frac{ay_{n}^{2}z_{n}}{m+y_{n}^{2}}-cy_{n},\\ z_{n+1}=z_{n}+\frac{aby_{n}^{2}z_{n}}{m+y_{n}^{2}}-dz_{n}, \end{array}\right.$$ (2)

where *x*_*n*_, *y*_*n*_, *z*_*n*_ ≥ 0 represent the densities of susceptible prey population Tilapia, infected prey population Tilapia and their predator population Pelican at time *n*, respectively. Here parameters *r*, *K*, *β*, *m*, *a*, *b*, *c*, *d* are all nonnegative constants and their biological meanings are given Table 1.

**Table 1:** Description and estimation of parameters in system (2).

Parameter	Description	value
*r*	the intrinsic growth rate of the prey population	[43]
*K*	the environment carrying capacity of the prey population	[8]
*β*	the transmission coefficient	[8]
*m*	the degree of capturing saturation	estimated
*a*	the predation coefficient	estimated
*b*	the conversion efficiency of the predator	[8]
*c*	the death rate of the infected prey	[8]
*d*	the death rate of the predator	[43]

Moreover,

$$\left(r,K,\beta ,m,a,b,c,d\right)\in \Omega ≔\{\left(r,K,\beta ,m,a,b,c,d\right)\in R_{+}^{8}:\;b,c,d\in \left(0,1\right)\}.$$

The rest of this paper is organized as follows. In Section 2, The boundedness of solutions of system (2) is given. In Section 3, we study the existence and stability of the fixed points. In Section 4, the transcritical bifurcation of boundary fixed points and the flip and Neimark-Sacker bifurcation of positive fixed points are analyzed. In Section 5, we use a hybrid control strategy to control the Neimark-Sacker bifurcation and the flip bifurcation. Finally, numerical simulations and conclusions are given in Section 6.

## Boundedness

For system (2) we always assume that all initial values are non-negative and all the parameters are positive. The boundedness of solutions of system (2) is given by the following lemma.

**Lemma 1** Let *μ* = *min*(*c*, *d*), then all the solutions of system (2) will lie in the region

$$\begin{array}{c} B≔\{\left(x_{n},y_{n},z_{n}\right)\in R_{+}^{3}:0\leq x_{n}+y_{n}+z_{n}\leq \frac{K(r+1)}{\mu }\} \end{array}$$

for all initial values *x*_0_, *y*_0_, *z*_0_ ≥ 0 as *n* → + ∞.

**Proof 1** For system (2), we always assume that *x*_0_, *y*_0_, *z*_0_ ≥ 0. Because the environmental carrying capacity of the prey population is *K*, therefore *x*_*n*_ ≤ *K*.

Let *x*_*n*_ + *y*_*n*_ + *z*_*n*_ = *M*_*n*_, adding all the equations of system (2), we get

$$\begin{array}{cc} M_{n+1}\;\;\;\; & =M_{n}+rx_{n}(1-\frac{x_{n}+y_{n}}{K})-\left(1-b\right)\frac{ay_{n}^{2}z_{n}}{m+y_{n}^{2}}-cy_{n}-dz_{n},\\ & \leq M_{n}+rx_{n}(1-\frac{x_{n}}{K})-cy_{n}-dz_{n},\\ & \leq M_{n}+\left(r+1\right)x_{n}-x_{n}-cy_{n}-dz_{n},\\ & \leq M_{n}+\left(r+1\right)K-\mu M_{n}, \end{array}$$ (3)

where *μ* = *min*{*c*, *d*}.

If there exists *l* ∈ *N* such that *M*_*l*+1_ > *M*_*l*_, then we get

$$\begin{array}{cc} & rx_{l}(1-\frac{x_{l}+y_{l}}{K})-\left(1-b\right)\frac{ay_{l}^{2}z_{l}}{m+y_{l}^{2}}-cy_{l}-dz_{l}\geq 0. \end{array}$$

Hence, we have that *M*_*l*_ ≤ M_*_, where M_*_ = (*r* + 1)*K*/*μ*. We claim that *M*_*n*_ ≤ M_*_ for all *n* ≥ *l*. Otherwise, we assume that there exists a *q* ∈ *N* such that *M*_*q*_ > M_*_, where *q* ≥ *l*. Let $\tilde{q}$ be the smallest integer such that $M_{\tilde{q}}>M_{*}$, then $M_{\tilde{q}-1}\leq M_{*}$. From inequality (3) we get $M_{\tilde{q}}\leq M_{*}$, which is a contradiction. Hence the claim is hold.

If *M*_*n*+1_ ≤ *M*_*n*_ for all *n* ∈ *N*. Let ${\text{lim}}_{n\to \infty }\;M_{n}=\bar{M}$, we claim that $\bar{M}\leq M_{*}$. Otherwise, we assume that $\bar{M}>M_{*}$. Taking limit of equation

$$\begin{array}{c} M_{n+1}=M_{n}+rx_{n}(1-\frac{x_{n}+y_{n}}{K})-\left(1-b\right)\frac{ay_{n}^{2}z_{n}}{m+y_{n}^{2}}-cy_{n}-dz_{n}, \end{array}$$

we get

$$\begin{array}{c} \underset{n\to \infty }{\text{lim}}\;(rx_{n}(1-\frac{x_{n}+y_{n}}{K})-\left(1-b\right)\frac{ay_{n}^{2}z_{n}}{m+y_{n}^{2}}-cy_{n}-dz_{n})=0. \end{array}$$

But

$$\begin{array}{cc} rx_{n}(1-\frac{x_{n}+y_{n}}{K})-\left(1-b\right)\frac{ay_{n}^{2}z_{n}}{m+y_{n}^{2}}-cy_{n}-dz_{n}\;\;\; & \leq rx_{n}(1-\frac{x_{n}}{K})-cy_{n}-dz_{n},\\ & \leq \left(r+1\right)K-\mu \bar{M},\\ & <0, \end{array}$$

for large enough *n* ∈ *N*, which is a contradiction. Hence the claim is proved. Thus, all the solutions of system (2) will lie in the region *B*.

## Existence of fixed points and stability

In this section, we give the sufficient and necessary conditions for the existence of fixed points for system (2), and analyze their properties.

Defined *R*_0_ = *βK*/*c*. Because *β* is the transmission coefficient, so *βK* represents the number of newly infected individuals when all the prey populations are susceptible at the beginning of the disease. For *c* is the death rate of infected prey because of natural death and disease induced mortality, so 1/*c* is the duration of infection of an infected prey. Therefore, *R*_0_ is the disease reproduction number in the prey.

Let

$$\begin{array}{cc} \Lambda_{1}≔ & \{\left(r,K,\beta ,m,a,b,c,d\right)\in \Omega :R_{0}>1,ab\leq d\},\\ \Lambda_{2}≔ & \{\left(r,K,\beta ,m,a,b,c,d\right)\in \Omega :R_{0}>1,ab>d,1<R_{0}\leq 1\\ & +\frac{\beta (r+\beta K)}{cr}\sqrt{\frac{dm}{ab-d}}\},\\ \Lambda_{3}≔ & \{\left(r,K,\beta ,m,a,b,c,d\right)\in \Omega :R_{0}>1,ab>d,R_{0}>1+\frac{\beta (r+\beta K)}{cr}\sqrt{\frac{dm}{ab-d}}\}. \end{array}$$ (4)

Firstly, on the sufficient and necessary conditions for the existence of the nonnegative fixed points of system (2), we give the the following theorem.

**Theorem 1** System (2) has only two fixed points *E*_0_(0, 0, 0) and *E*_1_(*K*, 0, 0) if and only if *R*_0_ ≤ 1, exactly three fixed points *E*_0_(0, 0, 0), *E*_1_(*K*, 0, 0) and $E_{2}\left(\hat{x},\hat{y},0\right)$ if and only if(*r*, *K*, *β*, *m*, *a*, *b*, *c*, *d*) ∈ Λ_1_ ∪ Λ_2_, where

$$\begin{array}{c} \hat{x}≔\frac{c}{\beta },\;\;\hat{y}≔\frac{r(\beta K-c)}{\beta (r+\beta K)},\;\; \end{array}$$ (5)

exactly four fixed points *E*_0_(0, 0, 0), *E*_1_(*K*, 0, 0), $E_{2}\left(\hat{x},\hat{y},0\right)$, *E*_3_(x_*_, y_*_, z_*_) if and only if (*r*, *K*, *β*, *m*, *a*, *b*, *c*, *d*) ∈ Λ_3_, where

$$\begin{array}{c} x_{*}≔K-(1+\frac{\beta K}{r})y_{*},\;\;y_{*}≔\sqrt{\frac{dm}{ab-d}},\;\;z_{*}≔\frac{bm(\beta x_{*}-c)}{\sqrt{dm(ab-d)}}, \end{array}$$ (6)

where Λ_*i*_’s (*i* = 1, 2, 3) are defined in (4).

**Proof 2** In Ω, the fixed points of system (2) satisfy

$$\left\{ \begin{array}{c} x_{n}=x_{n}+x_{n}(r(1-\frac{x_{n}+y_{n}}{K})-\beta y_{n}),\\ y_{n}=y_{n}+y_{n}(\beta x_{n}-\frac{ay_{n}z_{n}}{m+y_{n}^{2}}-c),\\ z_{n}=z_{n}+z_{n}(\frac{aby_{n}^{2}}{m+y_{n}^{2}}-d). \end{array}\right.$$ (7)

Clearly, (0, 0, 0) and (*K*, 0, 0) are always the solution of (7). Then we consider other possible solutions.

Since the second equation in (7) implies *y*_*n*_ = 0 if *x*_*n*_ = 0, the third equation in (7) implies *z*_*n*_ = 0 if *y*_*n*_ = 0. Therefore, for the boundary equilibrium point, we only need to consider the case of *x*_*n*_ ≠ 0, *y*_*n*_ ≠ 0, *z*_*n*_ = 0. If *x*_*n*_ ≠ 0, *y*_*n*_ ≠ 0, *z*_*n*_ = 0 and *R*_0_ ≤ 1, the second equation in (7) holds only if *y*_*n*_ = 0. Thus, there are only two solutions (0, 0, 0) and (*K*, 0, 0) of (7) when *R*_0_ ≤ 1. If *x*_*n*_ ≠ 0, *y*_*n*_ ≠ 0, *z*_*n*_ = 0 and *R*_0_ > 1, (7) has a solution $\left(\hat{x},\hat{y},0\right)$, where $\hat{x},\;\hat{y}$ are given in (5).

When *R*_0_ > 1, because $0<y_{n}^{2}/\left(m+y_{n}^{2}\right)<1$, from the third equation of (7) we can see that when *ab* ≤ *d*, the third equation of (7) holds only if *z*_*n*_ = 0. When *ab* > *d*, if *z*_*n*_ ≠ 0, from the third equation of (7), we can get $y_{n}=\sqrt{dm/(ab-d)}$. From the first equation of (7), we can get $x_{n}=K-\sqrt{dm/(ab-d)}\left(r+\beta K\right)/r$. If $1<R_{0}<1+\sqrt{dm/(ab-d)}\beta \left(r+\beta K\right)/\left(cr\right)$, the second equation of (7) not holds. If $R_{0}=1+\sqrt{dm/(ab-d)}\beta \left(r+\beta K\right)/\left(cr\right)$, the second equation of (7) holds only if *z*_*n*_ = 0. If *z*_*n*_ = 0, then *x*_*n*_ = 0, *y*_*n*_ = 0 or *x*_*n*_ = *K*, *y*_*n*_ = 0 or $x_{n}=\hat{x}$, $y_{n}=\hat{y}$. Thus, there are only three solutions (0, 0, 0), (*K*, 0, 0) and $\left(\hat{x},\hat{y},0\right)$ of (7) when (*r*, *K*, *β*, *m*, *a*, *b*, *c*, *d*) ∈ Λ_1_ ∪ Λ_2_.

When *ab* > *d* and $R_{0}>1+\sqrt{dm/(ab-d)}\beta \left(r+\beta K\right)/\left(cr\right)$, if *z*_*n*_ ≠ 0, from the third equation of (7), we can get $y_{n}=\sqrt{dm/(ab-d)}>0$. From the first equation of (7), we get

$$\begin{array}{c} x_{n}=K-(1+\frac{\beta K}{r})y_{n}=\frac{c}{\beta }(R_{0}-\frac{\beta (r+\beta K)}{cr}\sqrt{\frac{dm}{ab-d}})>0. \end{array}$$

From the second equation of (7), we get

$$\begin{array}{c} z_{n}=\frac{bm(\beta x_{n}-c)}{\sqrt{dm(ab-d)}}=\frac{bcm}{\sqrt{dm(ab-d)}}(R_{0}-(1+\frac{\beta (r+\beta K)}{cr}\sqrt{\frac{dm}{ab-d}}))>0. \end{array}$$

so (7) has a unique positive solution (x_*_, y_*_, z_*_), where x_*_, y_*_, z_*_ are given in (6). If *z*_*n*_ = 0, then *x*_*n*_ = 0, *y*_*n*_ = 0 or *x*_*n*_ = *K*, *y*_*n*_ = 0 or $x_{n}=\hat{x}$, $y_{n}=\hat{y}$. Thus, there are exactly four fixed points *E*_0_(0, 0, 0), *E*_1_(*K*, 0, 0), $E_{2}\left(\hat{x},\hat{y},0\right)$ and *E*_3_(x_*_, y_*_, z_*_) if and only if (*r*, *K*, *β*, *m*, *a*, *b*, *c*, *d*) ∈ Λ_4_.

For a discrete dynamical system on *R*^3^, let the Jacobian matrix of this system at a fixed point (*x*, *y*, *z*) be *J*(*x*, *y*, *z*). We denote the three eigenvalues of *J*(*x*, *y*, *z*) by λ_*i*_ (*i* = 1, 2, 3). We recall some concepts for a discrete dynamical system on *R*^3^ [47]. If |λ_*i*_| < 1 for all eigenvalues, then (*x*, *y*, *z*) is called a sink and is locally asymptotically stable; if |λ_*i*_| > 1 for some eigenvalues and |λ_*j*_| < 1 for the others, then (*x*, *y*, *z*) is called a saddle and is unstable; if |λ_*i*_| > 1 for all eigenvalues, then (*x*, *y*, *z*) is called a source and is unstable; if |λ_*i*_| = 1 for any eigenvalue, then (*x*, *y*, *z*) is called non-hyperbolic.

**Theorem 2** (1) The fixed point *E*_0_ is always unstable.

(2) For the fixed point *E*_1_, we have the following conclusions.

(a) If *R*_0_ < 1 and 0 < *r* < 2, *E*_1_ is locally asymptotically stable.

(b) If *R*_0_ > 1 or *r* > 2, *E*_1_ is unstable.

(c) If *R*_0_ = 1 or *r* = 2, *E*_1_ is non-hyperbolic.

**Proof 3** Because the Jacobian matrix of system (2) at *E*_0_ is given by

$$\begin{array}{c} J\left(E_{0}\right)=(\begin{array}{ccc} 1+r & 0 & 0\\ 0 & 1-c & 0\\ 0 & 0 & 1-d \end{array}), \end{array}$$

one of the eigenvalue of matrix *J*(*E*_0_) is λ_1_ = 1 + *r* > 1, so the fixed point *E*_0_ is always unstable.

The Jacobian matrix of system (2) at *E*_1_ is given by

$$\begin{array}{c} J\left(E_{1}\right)=(\begin{array}{ccc} 1-r & -(r+\beta K) & 0\\ 0 & \beta K-c+1 & 0\\ 0 & 0 & 1-d \end{array}). \end{array}$$

If *R*_0_ < 1 and 0 < *r* < 2, we get |λ_1_| = |1 − *r*| < 1, |λ_2_| = |*βK* − *c* + 1| < 1, |λ_3_| = 1 − *d* < 1. In this case, *E*_1_ is locally asymptotically stable. If *R*_0_ > 1 or *r* > 2, we get |λ_1_| = |1 − *r*| > 1 or |λ_2_| = |*βK* − *c* + 1| > 1, |λ_3_| = 1 − *d* < 1. Therefore, *E*_1_ is unstable in this case. If *R*_0_ = 1 or *r* = 2, we get λ_1_| = |1 − *r*| = 1 or |λ_2_| = |*βK* − *c* + 1| = 1, |λ_3_| = 1 − *d* < 1. Thus, *E*_1_ is non-hyperbolic in this case.

**Lemma 2** [48] Let *F*(λ) = λ^2^ + *B*λ + *C*, where *B* and *C* are constants. Suppose *F*(1) > 0 and λ_1_, λ_2_ are two roots of *F*(λ) = 0. Then

(1) |λ_1_| < 1 and |λ_2_| < 1 if and only if *F*(−1) > 0 and *C* < 1;
(2) |λ_1_| < 1 and |λ_2_| > 1 if and only if *F*(−1) < 0;
(3) |λ_1_| > 1 and |λ_2_| > 1 if and only if *F*(−1) > 0 and *C* > 1;
(4) λ_1_ = −1 and |λ_2_| ≠ 1 if and only if *F*(−1) = 0 and *B* ≠ 0, 2;
(5) λ_1_ and λ_2_ are a pair of conjugate complex roots and |λ_1_| = |λ_2_| = 1 if and only if |*B*| < 2 and *C* = 1.

Next, we consider the fixed point *E*_2_. The Jacobian matrix of system (2) at *E*_2_ is

$$\begin{array}{c} J\left(E_{2}\right)=(\begin{array}{ccc} 1-\frac{r\hat{x}}{K} & -(r+\beta K) & 0\\ \beta \hat{y} & 1 & -\frac{a{\hat{y}}^{2}}{m+{\hat{y}}^{2}}\\ 0 & 0 & 1-d+\frac{ab{\hat{y}}^{2}}{m+{\hat{y}}^{2}} \end{array}). \end{array}$$

The corresponding characteristic equation of *J*(*E*_2_) is

$$f\left(\lambda \right)=\left(\lambda -w_{1}\right)\left(\lambda^{2}+B_{E_{2}}\lambda +C_{E_{2}}\right),$$

where

$$\begin{array}{c} w_{1}=1-d+\frac{ab{\hat{y}}^{2}}{m+{\hat{y}}^{2}},\;B_{E_{2}}=\frac{rc}{\beta K}-2,\;C_{E_{2}}=\left(1-\frac{rc}{\beta K}\right)+r\left(\beta K-c\right). \end{array}$$

Let $F_{E_{2}}\left(\lambda \right)=\lambda^{2}+B_{E_{2}}\lambda +C_{E_{2}}$, for the fixed point *E*_2_, we have the following conclusions.

**Theorem 3** (1) When *ab* ≤ *d* and *R*_0_ > 1 or *ab* > *d* and $1<R_{0}<1+\sqrt{dm/(ab-d)}\beta \left(r+\beta K\right)/\left(cr\right)$, we get

(a) *E*_2_ is locally asymptotically stable if $F_{E_{2}}\left(-1\right)>0$ and $C_{E_{2}}<1$;

(b) *E*_2_ is unstable if $F_{E_{2}}\left(-1\right)<0$ or $F_{E_{2}}\left(-1\right)>0$ and $C_{E_{2}}>1$;

(c) *E*_2_ is non-hyperbolic if $F_{E_{2}}\left(-1\right)=0$ and $B_{E_{2}}\neq 0,2$ or $|B_{E_{2}}|<2$ and $C_{E_{2}}=1$.

(2) When *ab* > *d* and $R_{0}=1+\sqrt{dm/(ab-d)}\beta \left(r+\beta K\right)/\left(cr\right)$, *E*_2_ is non-hyperbolic.

(3) When *ab* > *d* and $R_{0}>1+\sqrt{dm/(ab-d)}\beta \left(r+\beta K\right)/\left(cr\right)$, *E*_2_ is unstable.

**Proof 4** Obviously, *f*(λ) has one eigenvalue *w*_1_. When *ab* ≤ *d* and *R*_0_ > 1, we have

$$\begin{array}{c} \frac{ab{\hat{y}}^{2}}{m+{\hat{y}}^{2}}-d<0, \end{array}$$

when *ab* > *d* and $1<R_{0}<1+\sqrt{dm/(ab-d)}\beta \left(r+\beta K\right)/\left(cr\right)$, we get

$$\begin{array}{c} \hat{y}=\frac{r(\beta K-c)}{\beta (r+\beta K)}<\sqrt{\frac{dm}{ab-d}}, \end{array}$$

also have

$$\begin{array}{c} \frac{ab{\hat{y}}^{2}}{m+{\hat{y}}^{2}}-d<0. \end{array}$$

Thus, we get 0 < *w*_1_ < 1, when *ab* ≤ *d* and *R*_0_ > 1 or *ab* > *d* and $1<R_{0}<1+\sqrt{dm/(ab-d)}\beta \left(r+\beta K\right)/\left(cr\right)$. Since $F_{E_{2}}\left(1\right)=r\left(\beta K-c\right)>0$, by the Lemma 2 we know that *E*_2_ is locally asymptotically stable if $F_{E_{2}}\left(-1\right)>0$ and $C_{E_{2}}<1$. *E*_2_ is unstable if $F_{E_{2}}\left(-1\right)<0$ or $F_{E_{2}}\left(-1\right)>0$ and $C_{E_{2}}>1$. *E*_2_ is non-hyperbolic if $F_{E_{2}}\left(-1\right)=0$ and $B_{E_{2}}\neq 0,2$ or $|B_{E_{2}}|<2$ and $C_{E_{2}}=1$.

When *ab* > *d* and $R_{0}=1+\sqrt{dm/(ab-d)}\beta \left(r+\beta K\right)/\left(cr\right)$, we get *w*_1_ = 1. Thus, *E*_2_ is non-hyperbolic.

When *ab* > *d* and $R_{0}>1+\sqrt{dm/(ab-d)}\beta \left(r+\beta K\right)/\left(cr\right)$, we get *w*_1_ > 1. Therefore, *E*_2_ is unstable.

**Lemma 3** (Jury-criterion [49]) For the equation λ^3^ + *a*_2_λ^2^ + *a*_1_λ + *a*_0_ = 0, all roots lie within the unit disk if and only if the following conditions

$$\begin{array}{c} |a_{2}+a_{0}|<1+a_{1},\;\;|a_{1}-a_{2}a_{0}|<|1-a_{0}^{2}\left|,\;\;\right|a_{0}|<1 \end{array}$$

are satisfied, where *a*_0_, *a*_1_, *a*_2_ are real numbers.

In the follows, we use Lemma 3 to analyze the stability of the positive fixed point *E*_3_.

**Theorem 4** When *E*_3_ exists, *E*_3_ is locally asymptotically stable if and only if the following inequations hold true

$$\begin{array}{c} |b_{2}+b_{0}|<1+b_{1},\;\;|b_{1}-b_{2}b_{0}|<|1-b_{0}^{2}\left|,\;\;\right|b_{0}|<1, \end{array}$$ (8)

where

$$\begin{array}{cc} & b_{2}≔\frac{rx_{*}}{K}+\frac{\left(ab-2d\right)\left(\beta x_{*}-c\right)}{ab}-3,\\ & b_{1}≔3-\frac{2rx_{*}}{K}+(\left(2d-ab\right)(2-\frac{rx_{*}}{K})+2d\left(ab-d\right))\frac{\beta x_{*}-c}{ab}+(\beta +\frac{r}{K})\beta x_{*}y_{*},\\ & b_{0}≔(\frac{rx_{*}}{K}-1)(1+\left(2d-ab+2abd-2d^{2}\right)\frac{\beta x_{*}-c}{ab})-(\beta +\frac{r}{K})\beta x_{*}y_{*}. \end{array}$$ (9)

**Proof 5** The Jacobian matrix of system (2) at *E*_3_ is

$$\begin{array}{c} J\left(E_{3}\right)=(\begin{array}{ccc} 1-\frac{rx_{*}}{K} & -(\beta +\frac{r}{K})x_{*} & 0\\ \beta y_{*} & 1+\frac{\left(\beta x_{*}-c\right)\left(y_{*}^{2}-m\right)}{m+y_{*}^{2}} & -\frac{d}{b}\\ 0 & \frac{2bm(\beta x_{*}-c)}{m+y_{*}^{2}} & 1 \end{array}). \end{array}$$

The corresponding characteristic equation of *J*(*E*_3_) is

$$\begin{array}{c} \lambda^{3}+b_{2}\lambda^{2}+b_{1}\lambda +b_{0}=0, \end{array}$$

where *b*_*i*_ (*i* = 0, 1, 2) are defined in (9). According to Lemma 3, we get that *E*_3_ is locally asymptotically stable if and only if condition (8) is satisfied.

## Bifurcations analysis

In this section, we investigate possible bifurcation of system (2).

### Transcritical bifurcation

In this subsection, we will give the transcritical bifurcation of system (2).

**Theorem 5** In Ω, system (2) has two transcritical bifurcation surfaces

$$\Omega_{1}≔\left\{\left(r,K,\beta ,m,a,b,c,d\right)\in \Omega :\;\beta K-c=0,\;r<2\right\}$$

and

$$\begin{array}{cc} \Omega_{2}≔\{\left(r,K,\beta ,m,a,b,c,d\right)\in \Omega : & \beta K-c-\frac{\beta (r+\beta K)}{r}\sqrt{\frac{dm}{ab-d}}=0,\\ & \frac{2rc}{\beta K}-4<r\left(\beta K-c\right)<\frac{rc}{\beta K},\;r<\frac{4\beta K}{c}\} \end{array}$$

for *E*_1_ and *E*_2_, respectively. That is, transcritical bifurcation happens for *E*_1_ when the parameter crosses Ω_1_ into Λ_1_ ∪ Λ_2_ and *E*_2_ appears near *E*_1_; transcritical bifurcation happens for *E*_2_ when the parameter crosses Ω_2_ into Λ_3_ and *E*_3_ appears near *E*_2_.

**Proof 6** From Theorem 2, we know that the eigenvalues for *E*_1_ are 1 − *r*, *βK* − *c* + 1 and 1 − *d*. In Ω_1_, we require *r* < 2 to ensure |1 − *r*| < 1. In this case, the unique non-hyperbolic case is exact *βK* − *c* + 1 = 1, which corresponds to transcritical bifurcation surface Ω_1_. For the critical case *βK* − *c* = 0, that is *R*_0_ = 1, system (2) has only two fixed points *E*_0_ and *E*_1_. It is not hard to check that

$$\{\beta K-c-\frac{\beta (r+\beta K)}{r}\sqrt{\frac{dm}{ab-d}}\}{|}_{R_{0}=1}=-(\beta +\frac{c}{r})\sqrt{\frac{dm}{ab-d}}<0,$$

which means that the parameter goes into Λ_1_ ∪ Λ_2_ when *R*_0_ increases from 1. Thus, the third fixed point *E*_2_ appears by Theorem 1, i.e., transcritical bifurcation happens for *E*_1_. We observe that *E*_2_ is sufficiently close to *E*_1_ when 0 < *R*_0_ − 1 ≪ 1.

When *E*_2_ exists, from Theorem 3, we know that one of the eigenvalues for *E*_1_ is *w*_1_. If $F_{E_{2}}\left(-1\right)>0$ and $C_{E_{2}}<1$, i.e., 2*rc*/(*βK*) − 4 < *r*(*βK* − *c*) < *rc*/(*βK*), *r* < 4*βK*/*c*, the modulus of the other two eigenvalues with *E*_2_ is less than 1. In this case, the unique non-hyperbolic case is exact *w*_1_ = 1, which corresponds to transcritical bifurcation surface Ω_2_. When the parameter crosses Ω_2_ into Λ_3_, the forth fixed point *E*_3_ appears by Theorem 1, i.e., transcritical bifurcation happens for *E*_2_. We observe that *E*_3_ is sufficiently close to *E*_2_ when $0<R_{0}-\left(1+\sqrt{dm/(ab-d)}\beta \left(r+\beta K\right)/\left(cr\right)\right)\ll 1$.

Let *G*(λ) = λ^3^ + *b*_2_λ^2^ + *b*_1_λ + *b*_0_ is the characteristic polynomial of *J*(*E*_3_). From Theorem 1, we know that when *E*_3_ exists, we have *ab* − *d* > 0 and βx_*_ − *c* > 0. Thus, we can judge that

$$\begin{array}{c} G\left(1\right)=1+b_{2}+b_{1}+b_{0}=(1+2d\left(ab-d\right)\frac{\beta x_{*}-c}{ab})\frac{rx_{*}}{K}+(\beta +\frac{r}{K})\beta x_{*}y_{*}>0. \end{array}$$

This indicates that a fold bifurcation not happens for *E*_3_ of system (2). Thus, in the following, we will investigate the flip bifurcation and Neimark-Sacker bifurcation for *E*_3_ of system (2).

### Flip bifurcation

In this subsection, we will discuss parametric conditions under which the unique positive fixed point of system (2) undergoes a flip bifurcations. For this purpose, an explicit criterion for flip bifurcation is implemented without finding the eigenvalues of the system. The criterion is formulated using a set of simple equalities or inequalities that consist of the coefficients of the characteristic equation derived from the Jacobian matrix. Next, let’s introduce this criterion first [50].

Consider an n-dimensional map *x*_*n*+1_ = *f*_*μ*_(*x*_*n*_), where *x*_*n*+1_, *x*_*n*_ ∈ *R*^*n*^ and *μ* ∈ *R* is a parameter. Assume that *f* has a fixed point *x*_0_ and the characteristic polynomial of an n-dimensional map *f*_*μ*_ at *x*_0_ takes the form

$$\begin{array}{c} P_{\mu }\left(\lambda \right)=\sigma_{0}\lambda^{n}+\sigma_{1}\lambda^{n-1}+\cdots +\sigma_{n-1}\lambda +\sigma_{n}, \end{array}$$

where *σ*_0_ = 1 and *σ*_*i*_ = *σ*_*i*_(*μ*, *v*) (*i* = 1, 2, ⋯, *n*), *μ* is the bifurcation parameter, and *v* is the control parameter or the other to be determined. Consider the sequence of determinants $\Delta_{0}^{\pm }\left(\mu ,v\right)=1$, $\Delta_{1}^{\pm }\left(\mu ,v\right),\cdots ,\Delta_{n}^{\pm }\left(\mu ,v\right)$, where

$$\begin{array}{c} \Delta_{i}^{\pm }\left(\mu ,v\right)=|(\begin{array}{ccccc} 1 & \sigma_{1} & \sigma_{2} & \cdots & \sigma_{i-1}\\ 0 & 1 & \sigma_{1} & \cdots & \sigma_{i-2}\\ 0 & 0 & 1 & \cdots & \sigma_{i-3}\\ \cdots & \cdots & \cdots & \cdots & \cdots \\ 0 & 0 & 0 & \cdots & 1 \end{array})\pm (\begin{array}{ccccc} \sigma_{n-i+1} & \sigma_{n-i+2} & \cdots & \sigma_{n-1} & \sigma_{n}\\ \sigma_{n-i+2} & \sigma_{n-i+3} & \cdots & \sigma_{n} & 0\\ \cdots & \cdots & \cdots & \cdots & \cdots \\ \sigma_{n-1} & \sigma_{n} & \cdots & 0 & 0\\ \sigma_{n} & 0 & \cdots & 0 & 0 \end{array})|, \end{array}$$

*i* = 1, ⋯, *n*.

**Lemma 4** [50] Assume that *f*_*μ*_ has a fixed point *x*_0_. A flip bifurcation takes place at *μ* = *μ*_0_ if and only if the following conditions (H1) Eigenvalue assignment: $P_{\mu_{0}}\left(-1\right)=0$, $\Delta_{n-1}^{\pm }\left(\mu_{0},v\right)>0$, $P_{\mu_{0}}\left(1\right)>0$ and $\Delta_{j}^{\pm }\left(\mu_{0},v\right)>0$ for *j* = *n* − 2, *n* − 4, ⋯, 1 (resp. 2), when *n* is odd (resp. even). (H2) Transversality condition:

$$\frac{\sum_{i=1}^{n}{\left(-1\right)}^{n-i}\sigma_{i}^{\prime }}{\sum_{i=1}^{n}\left(n-i+1\right){\left(-1\right)}^{\left(n-i\right)}\sigma_{i-1}}\neq 0,$$

are satisfied, where $\sigma_{i}^{\prime }$ stands for the derivative of *σ*_*i*_(*μ*) with respect to *μ* at *μ* = *μ*_0_.

When *n* = 3, and we choose *r* as the perturbation parameter, in the following theorem, we give the parametric conditions for the flip bifurcation takes place at *r* = *r*_0_ for *E*_3_ of system (2).

**Theorem 6** The fixed point *E*_3_(x_*_, y_*_, z_*_) of system (2) undergoes a flip bifurcation at *r* = *r*_0_ if the conditions

$$\begin{array}{cc} & 1-b_{2}+b_{1}-b_{0}=0,\;\;\;1+b_{2}+b_{1}+b_{0}>0,\;\;\;\;1-b_{1}+b_{0}\left(b_{2}-b_{0}\right)>0,\\ & 1+b_{1}-b_{0}\left(b_{0}+b_{2}\right)>0,\;\;\;\;\;\;\;1\pm b_{0}>0,\;\;\;\;\;\;\frac{b_{2}^{\prime }-b_{1}^{\prime }+{b_{0}}^{\prime }}{3-2b_{2}+b_{1}}\neq 0. \end{array}$$ (10)

are satisfied, where *b*_*i*_ (*i* = 0, 1, 2) are defined in (9), $b_{i}^{\prime }$ is derivative of *b*_*i*_(*r*) with respect to *r* at *r* = *r*_0_, and *r*_0_ is a possible real root of equation 1 − *b*_2_(*r*) + *b*_1_(*r*) − *b*_0_(*r*) = 0.

**Proof 7** For *n* = 3 and *r*_0_ is the perturbation parameter. According to the criterion introduced in Lemma 4, if the conditions (H1) and (H2) are satisfied, then a flip bifurcation occurs at *r*_0_ for system (2). That is

$$\begin{array}{c} G_{r}\left(-1\right)=1-b_{2}+b_{1}-b_{0}=0,\;\;\;\;\;\;\;\;\;\;G_{r}\left(1\right)=1+b_{2}+b_{1}+b_{0}>0,\\ \Delta_{2}^{-}\left(r\right)=1-b_{1}+b_{0}\left(b_{2}-b_{0}\right)>0,\;\;\;\;\;\;\Delta_{2}^{+}\left(r\right)=1+b_{1}-b_{0}\left(b_{0}+b_{2}\right)>0,\\ \Delta_{1}^{\pm }\left(r\right)=1\pm b_{0}>0,\;\;\;\;\;\;\;\;\;\;\;\;\;\;\;\;\;\;\;\frac{\sum_{i=1}^{n}{\left(-1\right)}^{n-i}b_{i}^{\prime }}{\sum_{i=1}^{n}{\left(-1\right)}^{n-i}\left(n-i+1\right)b_{i-1}}=\frac{b_{2}^{\prime }-b_{1}^{\prime }+{b_{0}}^{\prime }}{3-2b_{2}+b_{1}}\neq 0. \end{array}$$

Then we get the conditions (10).

### Neimark-Sacker bifurcation

In this subsection, we discuss parametric conditions under which the unique positive fixed point *E*_3_ of system (2) undergoes a Neimark-Sacker bifurcations. For this purpose, an explicit criterion for Neimark-Sacker bifurcation is implemented without finding the eigenvalues of the system. We state the explicit criterion as follow.

**Lemma 5** [51] If the following conditions (C1) Eigenvalue assignment: $P_{\mu_{0}}\left(1\right)>0$, ${\left(-1\right)}^{n}P_{\mu_{0}}\left(-1\right)>0$, $\Delta_{n-1}^{-}\left(\mu_{0},v\right)=0$, $\Delta_{n-1}^{+}\left(\mu_{0},v\right)>0$, $\Delta_{i}^{\pm }\left(\mu_{0},v\right)>0$, for *i* = *n* − 3, *n* − 5, ⋯, 1 (or 2), when *n* is even(or odd, resp.), (C2) Transversality condition: $d\Delta_{n-1}^{-}\left(\mu_{0},v\right)/d\mu \neq 0$, (C3) Non-resonance condition *cos*(2*π*/*l*)≠*φ* or resonance condition *cos*(2*π*/*l*) = *φ*, where *l* = 3, 4, 5 ⋯ and $\phi =1-0.5P_{\mu_{0}}\left(1\right)\Delta_{n-3}^{-}\left(\mu_{0},v\right)/\Delta_{n-2}^{+}\left(\mu_{0},v\right)$, are satisfied, then Neimark-Sacker bifurcation occurs at *μ*_0_ for map *f*_*μ*_.

When *n* = 3 and *r* is taken as the bifurcation parameter, we give the parametric conditions for the Neimark-Sacker bifurcation takes place at *r* = *r*_0_ for *E*_3_ of system (2) in the following.

**Theorem 7** The fixed point *E*_3_(x_*_, y_*_, z_*_) of system (2) undergoes a Neimark-Sacker bifurcation at *r* = *r*_0_ if the conditions

$$\begin{array}{c} 1-b_{1}+b_{0}\left(b_{2}-b_{0}\right)=0,1+b_{1}-b_{0}\left(b_{0}+b_{2}\right)>0,1+b_{2}+b_{1}+b_{0}>0,\\ 1-b_{2}+b_{1}-b_{0}>0,\frac{d(1-b_{1}+b_{0}\left(b_{2}-b_{0}\right))}{dr}\neq 0,\;\text{cos}\;(\frac{2\pi }{l})\neq 1-\frac{1+b_{2}+b_{1}+b_{0}}{2(1+b_{0})} \end{array}$$ (11)

are satisfied, where *b*_*i*_ (*i* = 0, 1, 2) are defined in (9), and *r*_0_ is a possible real root of equation 1 − *b*_1_(*r*) + *b*_0_(*r*)(*b*_2_(*r*) − *b*_0_(*r*)) = 0.

**Proof 8** For *n* = 3 and *r* is the perturbation parameter. According to the criterion introduced in Lemma 5, if the conditions (C1), (C2) and (C3) are satisfied, then Neimark-Sacker bifurcation occurs at *r*_0_ for system (2). That is

$$\begin{array}{c} \Delta_{2}^{-}\left(r\right)=1-b_{1}+b_{0}\left(b_{2}-b_{0}\right)=0,\;\;\;\;\Delta_{2}^{+}\left(r\right)=1+b_{1}-b_{0}\left(b_{2}+b_{0}\right)>0,\\ G_{r}\left(1\right)=1+b_{2}+b_{1}+b_{0}>0,\;\;\;\;\;\;\;\;{\left(-1\right)}^{3}G_{r}\left(-1\right)=1-b_{2}+b_{1}-b_{0}>0,\\ {\frac{d\Delta_{2}^{-}\left(r\right)}{dr}|}_{r=r_{0}}=\frac{d(1-b_{1}+b_{0}\left(b_{2}-b_{0}\right))}{dr}\neq 0,\;\;\;\text{cos}\;(\frac{2\pi }{l})\neq 1-\frac{G_{r}\left(1\right)\Delta_{0}^{-}\left(r\right)}{2\Delta_{1}^{+}\left(r\right)}. \end{array}$$

Then we get the conditions (11).

## Bifurcation control

In order to prevent the serious damage or even extinction of the population caused by infectious diseases, a stable positive fixed point may be needed to maintain the sustainable development of the eco-epidemiological system. That is, it is better for the positive fixed point *E*_3_ to be an asymptotically stable of system (2) if it exists. Therefore, we would like to take certain control measures to avoid the happening of bifurcations. For this purpose, in this section we provide for system (2) a hybrid control, which is based on feedback control strategy and parameter perturbation (see [52, 53]).

Corresponding to the system (2), we construct a controlled system

$$\left\{ \begin{array}{c} x_{n+1}=\theta (x_{n}+rx_{n}(1-\frac{x_{n}+y_{n}}{K})-\beta x_{n}y_{n})+\left(1-\theta \right)x_{n},\\ y_{n+1}=\theta (y_{n}+\beta x_{n}y_{n}-\frac{ay_{n}^{2}z_{n}}{m+y_{n}^{2}}-cy_{n})+\left(1-\theta \right)y_{n},\\ z_{n+1}=\theta (z_{n}+\frac{aby_{n}^{2}z_{n}}{m+y_{n}^{2}}-dz_{n})+\left(1-\theta \right)z_{n}. \end{array}\right.$$ (12)

where 0 < *θ* < 1, and *θ* is called a *control parameter*. Obviously, controlled system (12) is exactly (2) if *θ* = 1. System (12) has same fixed points as (2). The Jacobian matrix for (12) at *E*_3_(x_*_, y_*_, z_*_) is

$$\begin{array}{c} J_{con}=(\begin{array}{ccc} 1-\theta \frac{rx_{*}}{K} & -\theta (\beta +\frac{r}{K})x_{*} & 0\\ \theta \beta y_{*} & 1+\theta \frac{\left(\beta x_{*}-c\right)\left(y_{*}^{2}-m\right)}{m+y_{*}^{2}} & -\theta \frac{d}{b}\\ 0 & \theta \frac{2bm(\beta x_{*}-c)}{m+y_{*}^{2}} & 1 \end{array}). \end{array}$$

The corresponding characteristic equation of *J*_*con*_ is

$$\begin{array}{c} \lambda^{3}+c_{2}\lambda^{2}+c_{1}\lambda +c_{0}=0, \end{array}$$ (13)

where

$$\begin{array}{cc} c_{2}≔ & \theta \frac{rx_{*}}{K}+\theta \frac{\left(ab-2d\right)\left(\beta x_{*}-c\right)}{ab}-3,\\ c_{1}≔ & 3-2\theta \frac{rx_{*}}{K}+(\theta \left(2d-ab\right)(2-\theta \frac{rx_{*}}{K})+2d\left(ab-d\right))\frac{\beta x_{*}-c}{ab}\\ & +\theta^{2}(\beta +\frac{r}{K})\beta x_{*}y_{*},\\ c_{0}≔ & (\theta \frac{rx_{*}}{K}-1)(1+(\theta (2d-ab)+2d(ab-d))\frac{\beta x_{*}-c}{ab})-\theta^{2}(\beta +\frac{r}{K})\beta x_{*}y_{*}. \end{array}$$ (14)

Then by Lemma 3 we have the following theorem.

**Theorem 8** When *E*_3_ exists, the unique positive fixed point *E*_3_ of system (12) is locally asymptotically stable if and only if

$$\begin{array}{c} |c_{2}+c_{0}|<1+c_{1},\;\;|c_{1}-c_{2}c_{0}|<|1-c_{0}^{2}\left|,\;\;\right|c_{0}|<1, \end{array}$$ (15)

where *c*_*i*_ (*i* = 0, 1, 2) are defined in (14).

**Proof 9** Clearly, we only need to prove that the modulus of all eigenvalue is less than 1 for characteristic equation (13), which is equivalent to

$$\begin{array}{c} |c_{2}+c_{0}|<1+c_{1},\;\;|c_{1}-c_{2}c_{0}|<|1-c_{0}^{2}\left|,\;\;\right|c_{0}|<1, \end{array}$$

by the conditions given in Lemma 3, then we get the conclusion in this theorem.

## Numerical simulations and conclusions

In this section, we give the phase portraits, bifurcation diagrams, and Lyapunov exponents to illustrate our theoretical results numerically. We selected parameters based on the reference [8, 43] that studied the interaction between Pelican and Tilapia in the Salton Sea, and their biological meanings are stated in Table 1.

**Fig 2:**
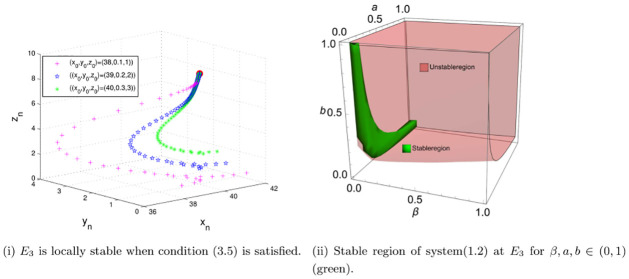
Local stability of *E*_3_.

Firstly, we show that *E*_3_ is locally stable when condition (8) is satisfied. Taking (*r*, *K*, *β*, *m*, *a*, *b*, *c*, *d*) = (1.8, 40, 0.006, 10, 0.5, 0.7655, 0.0019, 0.01) ∈ Λ_3_, which satisfy condition (8). We give the phase portraits of system (2) starting from (38, 0.1, 1), (39, 0.2, 2), (40, 0.3, 3), separately, in Fig 2(i). We can see that the trajectories of system (2) approach the fixed point *E*_3_(39.4, 0.5, 9.3). In Fig 2(ii), we give the stable region (Green region) of system (2) at *E*_3_ for *β*, *a*, *b* ∈ (0, 1) and other parameters are same as Fig 2(i). The red region is the area Λ_3_ where *E*_3_ exists. These are consistent to our Theorem 4.

**Fig 3:**
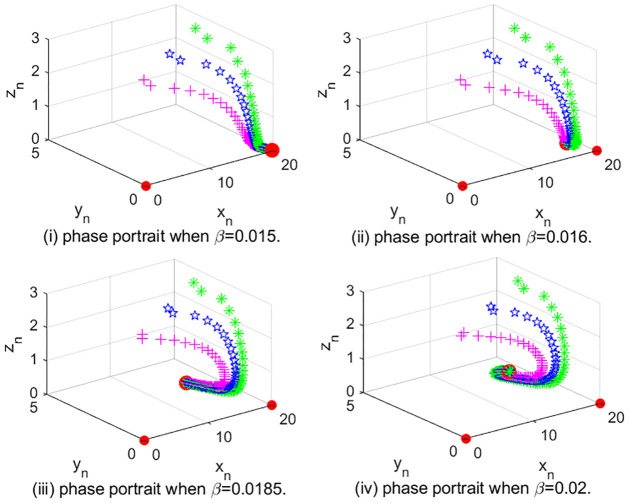
(i) and (ii) phase portraits for *β* in the small neighborhood of Ω_1_, (iii) and (iv) phase portraits for *β* in the small neighborhood of Ω_2_.

In Fig 3, we show the phase diagram of system (2) with different *β* values. Taking (*r*, *K*, *β*, *m*, *a*, *b*, *c*, *d*) = (1.8, 20, 0.015, 10, 0.5, 0.8, 0.3, 0.2) ∈ Ω_1_, we show the orbits starting from (15, 5, 1), (16, 4, 2), (17, 3, 3), separately, in Fig 3(i) and observe that system (2) has fixed point (0, 0, 0) and (*K*, 0, 0). (0, 0, 0) is always unstable, and (*K*, 0, 0) is locally asymptotically stable. When we change *β* from 0.015 to 0.016, we observe that another fixed point appears in Fig 3(ii). That is, transcritical bifurcation happens for *E*_1_ when the parameter crosses Ω_1_ into Λ_1_ ∪ Λ_2_. Taking (*r*, *K*, *β*, *m*, *a*, *b*, *c*, *d*) = (1.8, 20, 0.0185, 10, 0.5, 0.8, 0.3, 0.2) ∈ Ω_2_, we show the orbits starting from (15, 5, 1), (16, 4, 2), (17, 3, 3), separately, in Fig 3(iii) and observe that system (2) has three fixed points. When we change *β* from 0.0185 to 0.02, we observe that another fixed point appears in Fig 3(iv). That is, transcritical bifurcation happens for *E*_2_ when the parameter crosses Ω_2_ into Λ_3_. These are consistent to our Theorem 5.

**Fig 4:**
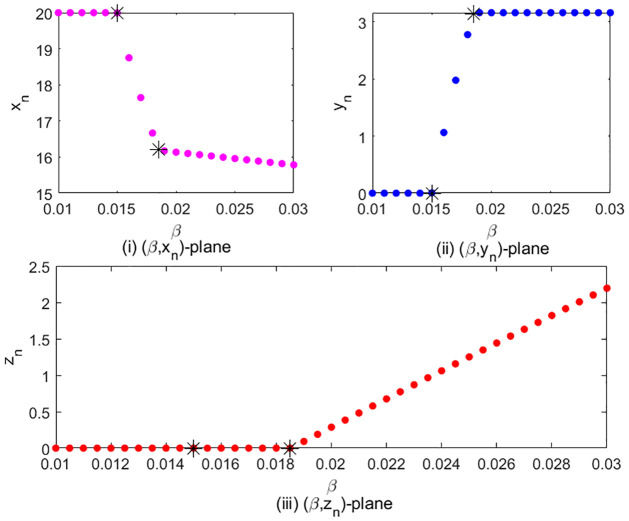
Transcritical bifurcation for *β* ∈ [0.012, 0.03].

In Fig 4, we show the twice transcritical bifurcations when changing *β* in [0.012, 0.03] and taking (*r*, *K*, *m*, *a*, *b*, *c*, *d*) = (1.8, 20, 10, 0.5, 0.8, 0.3, 0.2) with initial value (*x*_0_, *y*_0_, *z*_0_) = (16, 4, 1). In fact, bifurcation diagram shows the stable fixed point as *β* varies. In Fig 4, we iterate system (2) for 10000 times and observe the changes of the stable fixed point. In Fig 4(i), we observe that when *β* ≤ 0.015, the abscissa of the stable fixed point is 20, i.e. the value of *K*, which means that stable fixed point is *E*_1_ = (*K*, 0, 0) in this case. When *β* increases from 0.015, we can see that *x*_*n*_ starts to decrease. This is because the abscissa of fixed point *E*_2_ is $\hat{x}=c/\beta$, therefore, the *x* decreases as *β* increases. This means that another stable fixed point *E*_2_ emerges and the first transcritical bifurcation happens at *β* = 0.015. After *β* = 0.0185, the slope of the curve changes. This means the stable fixed point *E*_3_ emerges and the second transcritical bifurcation happens at *β* = 0.0185. The corresponding bifurcation diagrams for (*β*, *y*_*n*_) and (*β*, *z*_*n*_) are given in Fig 4(ii) and 4(iii) separately.

**Fig 5:**
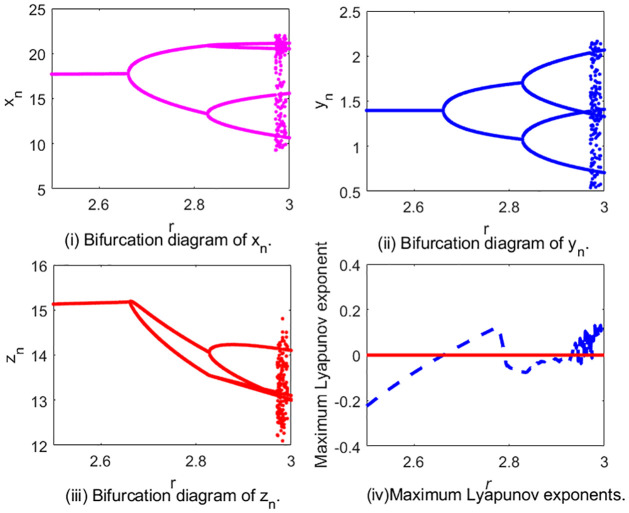
Flip bifurcation diagram of system (2).

In Fig 5, we show the flip bifurcation of system (2) by choosing *r* as the perturbation parameter. This diagram, which was obtained by changing *r* in [2.5, 3] and taking (*K*, *β*, *m*, *a*, *b*, *c*, *d*) = (20, 0.08, 10, 0.8, 0.7655, 0.0019, 0.1) with initial value (*x*_0_, *y*_0_, *z*_0_) = (17.76, 1.39, 15.17). When *r* = 2.66133, we get *P*_*r*_(−1) = 1 − *b*_2_ + *b*_1_ − *b*_0_ = 0, the characteristic equation of *J*(*E*_3_) is

$$\begin{array}{c} \lambda^{3}+0.31936\lambda^{2}-0.71933\lambda -0.03869=0. \end{array}$$ (16)

Eq (16) has a root λ_1_ = −1 and the other two roots are λ_2_ = 0.73340 and λ_3_ = −0.05275. So that we have |λ_2,3_| < 1. Further, we can verify that

$$\begin{array}{c} P_{r}\left(1\right)=1+b_{2}+b_{1}+b_{0}=0.56133>0,\;\;\Delta_{2}^{-}\left(r\right)=1-b_{1}+b_{0}\left(b_{2}-b_{0}\right)=1.70548>0,\\ \Delta_{2}^{+}\left(r\right)=1+b_{1}-b_{0}\left(b_{0}+b_{2}\right)=0.29152>0,\;\;\;\;\Delta_{1}^{+}\left(r\right)=1+b_{0}=0.96131>0,\\ \Delta_{1}^{-}\left(r\right)=1-b_{0}=1.03869>0,\;\;\;\;\;\;\;\;\;\;\;\;\;\;\;\;\;\;\;\;\;\;\frac{b_{2}^{\prime }-b_{1}^{\prime }+{b_{0}}^{\prime }}{3-2b_{2}+b_{1}}=1.18107\neq 0. \end{array}$$

Thus the conditions in Theorem 10 are satisfied. We get system (2) undergoes a flip bifurcation at *E*_3_. In Fig 5(i)–5(iii), we give the flip bifurcation diagrams for system (2) in the (*r*, *x*_*n*_)-plane, (*r*, *y*_*n*_)-plane and (*r*, *z*_*n*_)-plane. The Maximum Lyapunov exponents corresponding to Fig 5(i)–5(iii) are calculated and plotted in Fig 5(iv), which are consistent with the bifurcation diagram. For example, when *r* ∈ (2.5, 2.66133), the Maximum Lyapunov exponents are negative, which denote the system is stable. At *r* = 2.66133, the Maximum Lyapunov exponent is 0, which is corresponding to the flip bifurcation point. After that, we observe that some Lyapunov exponents are bigger than 0, some are smaller than 0, confirming the existence of the chaotic regions in the parametric space. In general the positive Lyapunov exponent is considered to be one of the characteristics implying the existence of chaos [54, 55].

**Fig 6:**
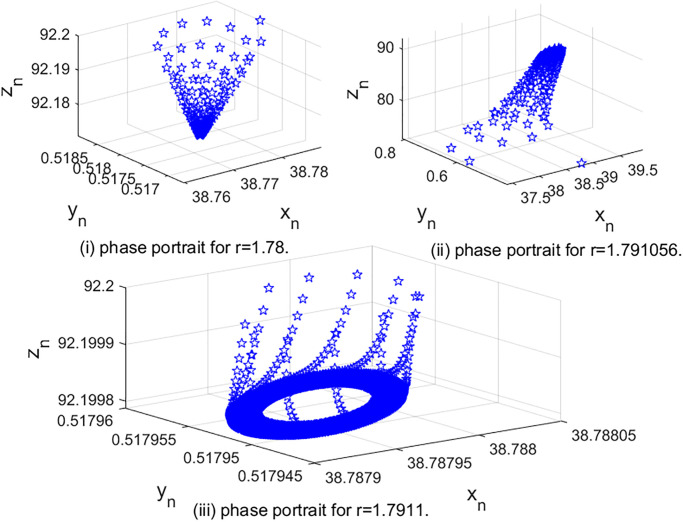
The phase portraits with various values of *r*.

In Fig 6, we show the Neimark-Sacker bifurcation of system (2) by choosing *r* as the perturbation parameter. By changing *r* in [1.7, 2.1], taking (*K*, *β*, *m*, *a*, *b*, *c*, *d*) = (40, 0.06, 10, 0.5, 0.7655, 0.0019, 0.01) with initial value (*x*_0_, *y*_0_, *z*_0_) = (38.788, 0.518, 92.1998). When *r* = 1.791065, we get $\Delta_{2}^{-}\left(r\right)=1-b_{1}+b_{0}\left(b_{2}-b_{0}\right)=0$, the characteristic equation of *J*(*E*_3_) is

$$\begin{array}{c} \lambda^{3}+0.94067\lambda^{2}-0.88207\lambda -0.97994=0, \end{array}$$

which has a root λ_1_ = 0.97994 and a pair of conjugate complex roots λ_2,3_ = −0.96030 ± 0.27897*i*, and |λ_2,3_| = 1. Further, we get that

$$\begin{array}{c} \Delta_{2}^{+}\left(1.791065\right)\;=\;1\;+\;b_{1}\;-\;b_{0}\left(b_{0}\;+\;b_{2}\right)\;=\;0.07945\;>\;0,\\ P_{1.791065}\left(1\right)=1\;+\;b_{2}\;+\;b_{1}\;+\;b_{0}\;=\;0.07866\;>\;0,\\ {\left(\;-1\;\right)}^{3}\;P_{1.791065}\left(\;-1\;\right)\;=\;1\;-\;b_{2}\;+\;b_{1}\;-\;b_{0}\;=\;0.15720\;>\;0,\\ {\frac{d(1\;-\;b_{1}\;+\;b_{0}\left(b_{2}\;-\;b_{0}\right))}{dr}|}_{r=1.791065}\;\;=\;-4.67030\;\neq \;0, \end{array}$$

and

$$\begin{array}{c} 1-\frac{P_{1.791065}\left(1\right)\Delta_{0}^{-}\left(1.791065\right)}{2\Delta_{1}^{+}\left(1.791065\right)}=1-\frac{1+b_{2}+b_{1}+b_{0}}{2(1+b_{0})}=-0.96030. \end{array}$$

From cos(2*π*/*l*) = −0.96030, we can obtain that *l* = 2.1978. So the non-resonance condition is satisfied. In Fig 6(i) and 6(ii), we observe that when *r* ≤ 1.791065, *E*_3_(x_*_, y_*_, z_*_) is stable. In Fig 6(iii), we can see when *r* > 1.791065, *E*_3_(x_*_, y_*_, z_*_) becomes unstable, and an attractive closed invariant curve appears. In this case, we get system (2) undergoes a Neimark-Sacker bifurcation at *E*_3_. This is consistent with Theorem 11.

**Fig 7:**
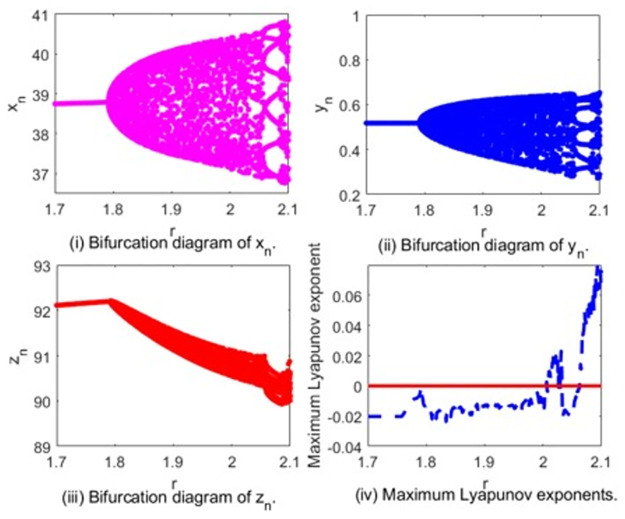
Neimark-Sacker bifurcation diagram of system (2) and maximum Lyapunov exponents corresponding to (i-iii).

In Fig 7, we give the Neimark-Sacker bifurcation diagrams for system (2) in the (*r*, *x*_*n*_)-plane, (*r*, *y*_*n*_)-plane and (*r*, *z*_*n*_)-plane. The Maximum Lyapunov exponents corresponding to Fig 7(i)–7(iii) are calculated and plotted in Fig 7(iv), which are consistent with the bifurcation diagram. When *r* ∈ (1.7, 1.791065), the Maximum Lyapunov exponents are negative, which denote the system is stable. At *r* = 1.791065, the Maximum Lyapunov exponent is 0, which is corresponding to the Neimark-Sacker bifurcation point. After that, we observe that some Lyapunov exponents are positive and some are negative, confirming the existence of the chaotic regions in the parametric space.

**Fig 8:**
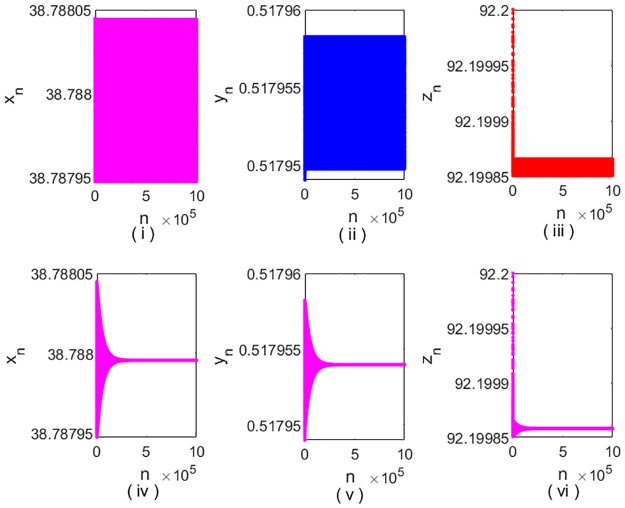
Time-series graph for system (2) and (12).

In Fig 8, we show the hybrid control method is effective in controlling Neimark-Sacker bifurcation for system (2). We take (*r*, *K*, *β*, *m*, *a*, *b*, *c*, *d*,) = (1.791065, 40, 0.06, 10, 0.5, 0.7655, 0.0019, 0.01) with an initial value of (38.788, 0.518, 92.1998). From Fig 6, we know that in this case, system (2) undergoes a Neimark-Sacker bifurcation at *E*_3_. We give the corresponding time-series graph in Fig 8(i)–8(iii) for system (2). In this case, from the condition (15), we get

$$\begin{array}{cc} Inequ1: & |-3.95397\theta^{2}+7.95999\theta -4.04529|\\ & <3.95397\theta^{2}-7.88133\theta +4.04529,\\ Inequ2: & |15.58\theta^{3}-23.75\theta^{2}+8.30\theta -0.09|\\ & <|-15.63\theta^{4}+31.79\theta^{3}-24.42\theta^{2}+8.40\theta -0.09|,\\ Inequ3: & |-3.95397\theta^{2}+4.01933\theta -1.04529|<1. \end{array}$$

**Fig 9:**
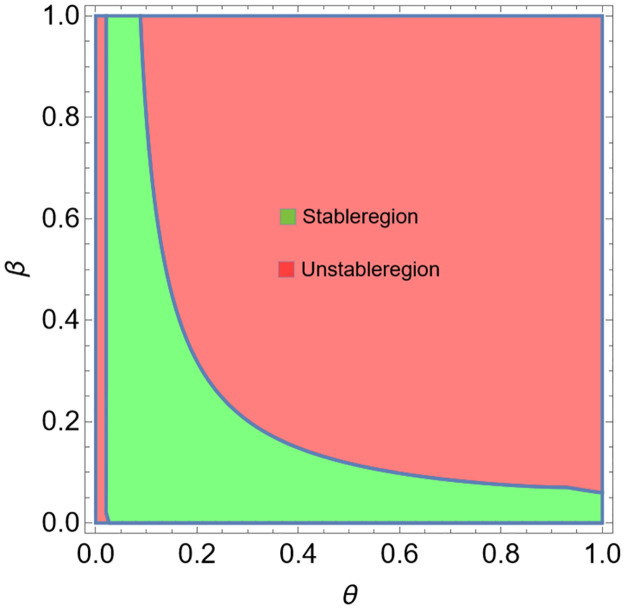
Stable region of system (12) when *β*, *θ* ∈ (0, 1).

According to Theorem 8, we know that when condition (15) is satisfied, we can control *E*_3_ to be a stable fixed point. After a simple calculation, we obtained that *E*_3_ is stable when 0.0205565 < *θ* < 1. Thus, we take *θ* = 0.99999, and give the time-series graph in Fig 8(iv)–8(vi) for system (12). We observe that the Neimark-Sacker bifurcation of system (2) is controlled effectively. We give the stable region of system (12) for *β*, *θ* ∈ (0, 1) in Fig 9.

In this paper, we have constructed the mathematical model to describe an interaction between Tilapia as a prey, Pelicans as predator, and botulism type C as the cause of disease in Tilapia. We discuss the dynamical behaviors of the system (2), establish that the system solution is bounded and get that the system has at most four fixed point depending on values of the system parameters. We obtained a threshold value *R*_0_ = *βK*/*c* such that *R*_0_ ≤ 1 leads to the complete disappearance of infected prey from the ecosystem and the eradication of the disease from the prey population. If the death rate of infected prey *c* is high and transmission rate from uninfected prey to infected prey *β* is low, then the chance of disease eradication from the ecosystem will be high. This means that we can control the spread of the disease by killing botulism type C with drugs. The local asymptotic stability of different fixed points are discussed here. The fixed point *E*_0_(0, 0, 0) is always unstable, which implies that the system can never be collapsed for any values of the system parameters. The fixed point *E*_1_(*K*, 0, 0) and $E_{2}\left(\hat{x},\hat{y},0\right)$ are locally asymptotically stable under some parametric restrictions. There exists a set of values of the system parameters for which the positive fixed point *E*_3_(x_*_, y_*_, z_*_) is locally asymptotically stable, i.e. both the populations can survive with positive density level. We notice that the stability of the fixed points is greatly influenced by the model parameters. We show that under certain parametric conditions system (2) undergoes transcritical bifurcations at boundary fixed points *E*_1_ and *E*_2_, using bifurcation theory. Further, by the explicit criteria for a flip bifurcation and a Neimark-Sacker bifurcation, we prove that the system undergoes both flip and Neimark-Sacker bifurcations at the fixed point *E*_3_ under some parametric conditions. From the ecological point of view, flip bifurcation is associated with the emergence of chaotic behavior, demonstrating the evolution of the prey and predator populations. An invariant curve bifurcates from the fixed point, meaning that predator and prey can coexist in a stable way and reproduce their densities. The dynamics on the invariant curve may be either periodic or quasi-periodic [40]. The Maximum Lyapunov exponents are numerically computed to confirm further the complexity of the dynamical behavior. Finally, we use the hybrid method to control the Neimark-Sacker bifurcation at fixed point *E*_3_. The result indicate that the nonlinear dynamics of such eco-epidemiology model not only depend on more bifurcation parameters but also are very sensitive to parameter perturbations, which are important for the control of biological species or infectious diseases. Finally, numerical simulations are carried out to confirm the validity of the theories and the effectiveness of the control method.

## Supporting information

S1 Data (XLSX)

S1 File (DOCX)
