# Supplementary material for: Bifurcation and hybrid control of a discrete eco-epidemiological model with Holling type-III
Source: PLoS One. 2024 Jul 18;19(7):e0304171. doi: 10.1371/journal.pone.0304171 (PMC11257352; doi:10.1371/journal.pone.0304171)
Supplement: S1 File — (DOCX) [file pone.0304171.s002.docx]

Matlab codes

Fig.2

function [R,F,P]=ecophase(r,k,bt,m,a,b,c,d,R0,F0,P0,n)

R=zeros(1,n);

F=zeros(1,n);

P=zeros(1,n);

R(1)=R0;

F(1)=F0;

P(1)=P0;

for j=1:n

R(j+1)=R(j)+r.*R(j).*(1-(R(j)+F(j))./k)-bt.*R(j).*F(j);

F(j+1)=F(j)+bt.*R(j).*F(j)-a.*F(j).^2.*P(j)./(m+F(j).^2)-c.*F(j);

P(j+1)=P(j)+a.*b.*F(j).^2.*P(j)./(m+F(j).^2)-d.*P(j);

end

plot3(R, F, P, 'bp');

%hold on

%plot3(R(n+1), F(n+1), P(n+1), 'r.','markersize',30);

xlabel('x_n','FontSize',15)

ylabel('y_n','FontSize',15)

zlabel('z_n','FontSize',15)

grid on

end

Fig.3

clear all

r=1.8;

k=40;

bt=0.0075;

m=10;

a=0.5;

b=0.7655;

c=0.3;

d=0.2;

R0=37;

F0=0.4;

P0=1;

n=1000;

R=zeros(1,n);

F=zeros(1,n);

P=zeros(1,n);

R(1)=R0;

F(1)=F0;

P(1)=P0;

for j=1:n

R(j+1)=R(j)+r.*R(j).*(1-(R(j)+F(j))./k)-bt.*R(j).*F(j);

F(j+1)=F(j)+bt.*R(j).*F(j)-a.*F(j).^2.*P(j)./(m+F(j).^2)-c.*F(j);

P(j+1)=P(j)+a.*b.*F(j).^2.*P(j)./(m+F(j).^2)-d.*P(j);

end

plot3(R, F, P, 'm+');

hold on

r=1.8;

k=40;

bt=0.0075;

m=10;

a=0.5;

b=0.7655;

c=0.3;

d=0.2;

R0=38;

F0=0.5;

P0=2;

n=1000;

R=zeros(1,n);

F=zeros(1,n);

P=zeros(1,n);

R(1)=R0;

F(1)=F0;

P(1)=P0;

for j=1:n

R(j+1)=R(j)+r.*R(j).*(1-(R(j)+F(j))./k)-bt.*R(j).*F(j);

F(j+1)=F(j)+bt.*R(j).*F(j)-a.*F(j).^2.*P(j)./(m+F(j).^2)-c.*F(j);

P(j+1)=P(j)+a.*b.*F(j).^2.*P(j)./(m+F(j).^2)-d.*P(j);

end

plot3(R, F, P, 'bp');

hold on

r=1.8;

k=40;

bt=0.0075;

m=10;

a=0.5;

b=0.7655;

c=0.3;

d=0.2;

R0=39;

F0=0.6;

P0=3;

n=1000;

R=zeros(1,n);

F=zeros(1,n);

P=zeros(1,n);

R(1)=R0;

F(1)=F0;

P(1)=P0;

for j=1:n

R(j+1)=R(j)+r.*R(j).*(1-(R(j)+F(j))./k)-bt.*R(j).*F(j);

F(j+1)=F(j)+bt.*R(j).*F(j)-a.*F(j).^2.*P(j)./(m+F(j).^2)-c.*F(j);

P(j+1)=P(j)+a.*b.*F(j).^2.*P(j)./(m+F(j).^2)-d.*P(j);

end

plot3(R, F, P, 'g*');

hold on

plot3(0, 0, 0, 'r.','markersize',20);

hold on

plot3(k, 0, 0, 'r.','markersize',20);

hold on

plot3(R(j),F(j),P(j), 'r.','markersize',30);

xlabel('x_n','FontSize',15)

ylabel('y_n','FontSize',15)

zlabel('z_n','FontSize',15)

legend('(x_0,y_0,z_0)=(40,0.1,2))','((x_0,y_0,z_0)=(39,0.2,1))','((x_0,y_0,z_0)=(41,0.15,3))')

grid on

Fig.4

function [R,F,P]= ecoflip(k,beta,m,a,b,c,d,R0,F0,P0,n)

R=zeros(1,n);

F=zeros(1,n);

P=zeros(1,n);

R(1)=R0;

F(1)=F0;

P(1)=P0;

for r=2.5:0.001:3

for j=1:n

R(j+1)=R(j)+R(j).*(r.*(1-(R(j)+F(j))./k)-beta.*F(j));

F(j+1)=F(j)+F(j).*(beta.*R(j)-a.*F(j).*P(j)./(m+F(j).*F(j))-c);

P(j+1)=P(j)+P(j).*(a.*b.*F(j).*F(j)./(m+F(j).*F(j))-d);

end

plot(r.*ones(1,10),R(n-10+1:n),'m.');

hold on

end

xlabel('r','FontSize',15)

ylabel('x_n','FontSize',15)

end

function [R,F,P]= ecofold3(r,k,m,a,b,c,d,R0,F0,P0,n)

R=zeros(1,n);

F=zeros(1,n);

P=zeros(1,n);

R(1)=R0;

F(1)=F0;

P(1)=P0;

for beta=0.012:0.0005:0.03

for j=1:n

R(j+1)=R(j)+R(j).*(r.*(1-(R(j)+F(j))./k)-beta.*F(j));

F(j+1)=F(j)+F(j).*(beta.*R(j)-a.*F(j).*P(j)./(m+F(j).*F(j))-c);

P(j+1)=P(j)+P(j).*(a.*b.*F(j).*F(j)./(m+F(j).*F(j))-d);

end

plot(beta.*ones(1,10),R(n-10+1:n),'m.','markersize',20);

hold on

end

plot( 0.015, 20, 'k*','markersize',15);

plot( 0.0185, 16.22, 'k*','markersize',15);

xlabel('\beta','FontSize',15)

ylabel('x_n','FontSize',15)

end

Fig.5

function [R,F,P]= ecoflip(k,beta,m,a,b,c,d,R0,F0,P0,n)

R=zeros(1,n);

F=zeros(1,n);

P=zeros(1,n);

R(1)=R0;

F(1)=F0;

P(1)=P0;

for r=2.5:0.001:3

for j=1:n

R(j+1)=R(j)+R(j).*(r.*(1-(R(j)+F(j))./k)-beta.*F(j));

F(j+1)=F(j)+F(j).*(beta.*R(j)-a.*F(j).*P(j)./(m+F(j).*F(j))-c);

P(j+1)=P(j)+P(j).*(a.*b.*F(j).*F(j)./(m+F(j).*F(j))-d);

end

plot(r.*ones(1,10),R(n-10+1:n),'m.');

hold on

end

xlabel('r','FontSize',15)

ylabel('x_n','FontSize',15)

end

Fig.6

function [R,F,P]= ecoNSx(k,beta,m,a,b,c,d,R0,F0,P0,n)

R=zeros(1,n);

F=zeros(1,n);

P=zeros(1,n);

R(1)=R0;

F(1)=F0;

P(1)=P0;

for r=1.7:0.001:2.1

for j=1:n

R(j+1)=R(j)+R(j).*(r.*(1-(R(j)+F(j))./k)-beta.*F(j));

F(j+1)=F(j)+F(j).*(beta.*R(j)-a.*F(j).*P(j)./(m+F(j).*F(j))-c);

P(j+1)=P(j)+P(j).*(a.*b.*F(j).*F(j)./(m+F(j).*F(j))-d);

end

plot(r.*ones(1,10),P(n-10+1:n),'r.','markersize',20);

hold on

end

xlabel('r','FontSize',15)

ylabel('z_n','FontSize',15)

axis([1.7 2.1 89.5 92.5 ])

end

Fig.7

function [T,R,F]= xT(r,k,beta,m,a,b,c,d,R0,F0,P0,n)

T=0:n;

R=zeros(1,n);

F=zeros(1,n);

P=zeros(1,n);

R(1)=R0;

F(1)=F0;

P(1)=P0;

for j=1:n

R(j+1)=R(j)+R(j).*(r.*(1-(R(j)+F(j))./k)-beta.*F(j));

F(j+1)=F(j)+F(j).*(beta.*R(j)-a.*F(j).*P(j)./(m+F(j).*F(j))-c);

P(j+1)=P(j)+P(j).*(a.*b.*F(j).*F(j)./(m+F(j).*F(j))-d);

end

plot(T, F, 'b.');

xlabel ('Time n ','fontsize',15);

ylabel ('y_n','fontsize',15);

end

Fig.8
